# Mouse Gastric Epithelial Cells Resist CagA Delivery by the *Helicobacter pylori* Type IV Secretion System

**DOI:** 10.3390/ijms23052492

**Published:** 2022-02-24

**Authors:** Rejina Shrestha, Naoko Murata-Kamiya, Satoshi Imai, Masami Yamamoto, Tetsuya Tsukamoto, Sachiyo Nomura, Masanori Hatakeyama

**Affiliations:** 1 Division of Microbiology, Graduate School of Medicine, The University of Tokyo, 7-3-1 Hongo, Bunkyo-ku, Tokyo 113-0033, Japan; rejinasth6@gmail.com (R.S.); naokokam@m.u-tokyo.ac.jp (N.M.-K.); isatoshi@m.u-tokyo.ac.jp (S.I.); 2 Division of Physiological Pathology, Department of Applied Science, School of Veterinary Nursing and Technology, Nippon Veterinary and Life Science University, 1-7-1 Kyonan-cho, Musashino-shi, Tokyo 180-8602, Japan; masami@nvlu.ac.jp; 3 Department of Diagnostic Pathology, Fujita Health University School of Medicine, 1-98 Dengakugakubo, Kutsukake-cho, Toyoake 470-1192, Japan; ttsukamt@fujita-hu.ac.jp; 4 Department of Gastrointestinal Surgery, Graduate School of Medicine, The University of Tokyo, 7-3-1 Hongo, Bunkyo-ku, Tokyo 113-0033, Japan; snomura-gi@umin.ac.jp

**Keywords:** *Helicobacter pylori*, mouse gastric epithelial cells, CEACAM, CagA, HopQ

## Abstract

The initial step in bacterial infection is adherence of the bacterium to the target cell surface. *Helicobacter pylori* exploits the interaction of bacterial adhesin protein HopQ with human epithelial CEACAMs (CEACAM1, 5, and 6) to stably adhere to gastric epithelial cells, which is necessary for delivery of the *H. pylori* CagA oncoprotein into the epithelial cells via a type IV secretion system. In contrast to human CEACAMs, however, HopQ does not interact with Ceacam1 (mouse CEACAM1) in vitro or in CHO cells ectopically expressing Ceacam1. Since the mouse genome lacks *Ceacam5* and *Ceacam6*, no significant HopQ–Ceacam interaction may occur in mouse gastric epithelial cells. Here, we found that the mouse stomach has a much lower expression level of Ceacam1 than the expression level of CEACAM1 in the human stomach. Consistently, mouse gastric epithelial cells resist CagA delivery by *cagA*-positive *H. pylori*, and the delivery is restored by ectopic expression of human CEACAM1 or CEACAM5 in mouse gastric epithelial cells. Thus, despite the fact that mice are routinely used for *H. pylori* infection studies, a low expression level of Ceacam1 in the mouse stomach together with the loss or greatly reduced interaction of HopQ with Ceacams make the mouse an inappropriate model for studying the role of *H. pylori*-delivered CagA in gastric pathogenesis, including the development of gastric cancer.

## 1. Introduction

Chronic infection with *Helicobacter pylori* plays an etiologic role in most if not all human gastric cancers, the third leading cause of cancer-related deaths worldwide, making the gastric pathogen the strongest risk factor for the development of gastric cancer [1,2]. *H. pylori* is classified into two major subgroups depending on the presence or absence of the *cagA* gene, which is located on the *cag* pathogenicity island (*cag*PAI), an approximately 40 kbp DNA segment that encodes the type IV secretion system (T4SS) [3], the bacterial microsyringe through which the *cagA*-encoded CagA protein is delivered into the attached gastric epithelial cells [4,5,6]. Infection with *cagA*-positive strains has been considered to be responsible for the development of gastric cancer at least in part by injecting CagA into gastric epithelial cells [7,8,9]. Once delivered inside the host cells, CagA is tethered to the inner plasma membrane where it acts as a protein scaffold that promiscuously interacts with multiple host proteins such as the oncogenic phosphatase SHP2 and polarity-regulating kinase PAR1b and perturbs their physiological functions, thereby contributing to the neoplastic transformation of CagA-delivered gastric epithelial cells [10,11,12,13,14,15,16]. Consistent with this, transgenic expression of CagA in mice gave rise to spontaneous gastrointestinal and hematological malignancies, confirming the oncogenic potential of the bacterial CagA protein in mammals [17].

The attachment of *H. pylori* to the human gastric epithelial cell surface involves BabA and SabA adhesins, which are outer membrane proteins (OMPs) that bind to Lewis b (Leb) and sialyl-Lewis x (sLex) antigens, respectively [18,19]. *H. pylori*-mediated CagA translocation is initiated by the interaction of CagA with host membrane phosphatidylserine and is facilitated by β1 integrin interaction of *cag*PAI-encoded T4SS components including CagL, CagA, CagY, and CagI [20,21]. Furthermore, recent studies have revealed that the *H. pylori* HopQ adhesin protein interacts with the amino-terminal immunoglobulin-like domain of human carcinoembryonic antigen-related cell adhesion molecules (CEACAMs) with high affinity [22,23], and this strong interaction is indispensable for *H. pylori*-mediated CagA delivery.

Human CEACAMs comprise 12 members of immunoglobulin-related glycoproteins. Some members such as CEACAM1, CEACAM3, and CEACAM4 have a transmembrane domain followed by a cytoplasmic domain and others such as CEACAM5, CEACAM6, CEACAM7 and CEACAM8 are anchored to the cell membrane via a glycosylphosphatidylinositol (GPI) linkage. CEACAMs display different expression patterns on different cell types and mediate multiple cellular processes including cell adhesion, differentiation, proliferation, motility, survival, and immune responses, through homophilic and/or heterophilic interactions with other molecules to transmit signals into the cells [24,25,26]. Several CEACAMs also serve as adhesions for bacteria and/or viruses, playing an important role in the clearance of microbial pathogens by innate immunity [24]. Gastric epithelial cells express the transmembrane receptor CEACAM1 as well as GPI-anchored CEACAM5 and CEACAM6 [22,23]. Notably, the CEACAM family has expanded considerably during primate evolution; only a few CEACAM orthologues are present in rodents and rodents do not possess GPI-anchored membrane Ceacams (mouse CEACAM) [25,26,27]. *H. pylori* HopQ strongly binds to the N-terminal IgV-like domain of human CEACAM1, 3, 5, and 6 with high affinity in vitro (*K*_D_~100 nM), but not CEACAM8, and among these, CEACAM1, 5, and 6 are expressed in various tissues/organs including the stomach epithelium (hereafter denoted here as epithelial CEACAMs) [22,23], while CEACAM3 and 8 are specifically expressed in hematopoietic cells [26]. The binding of HopQ to CEACAMs is crucial for *H. pylori*-mediated CagA delivery into human gastric epithelial cells, indicating that CEACAM1, 5, and 6 are involved in the process. Furthermore, the HopQ–CEACAM interaction is species-specific, since HopQ did not significantly bind to Ceacam1 (mouse CEACAM1) [22,23].

Although mice are not natural *H. pylori* hosts, mice have been used in many studies for artificial infection with *H. pylori cagA*-positive strains to study *H. pylori*-mediated gastric diseases including gastric cancer. Although infection of mice with *cagA*-positive strains gave rise to more severe mucosal damage than infection with *cagA*-negative strains did, there has been no report of induction of gastric cancer in mice with long-term *cagA*-positive *H. pylori* infection. In contrast, transgenic expression of CagA in mice gave rise to the spontaneous development of gastrointestinal and hematological malignancies [17]. These findings raise the possibility that *cagA*-positive *H. pylori* cannot efficiently deliver CagA into mouse gastric epithelial cells due to lack of Ceacams that bind *H. pylori* HopQ. In the present study, we examined the ability of *cagA*-positive *H. pylori* to deliver CagA into mouse gastric epithelial cells in the context of HopQ–Ceacam interactions.

## 2. Results

### 2.1. CagA-Positive Helicobacter pylori Is Capable of Delivering CagA into Human Gastric Epithelial Cells but Not Mouse Gastric Epithelial Cells

**Figure 1:**
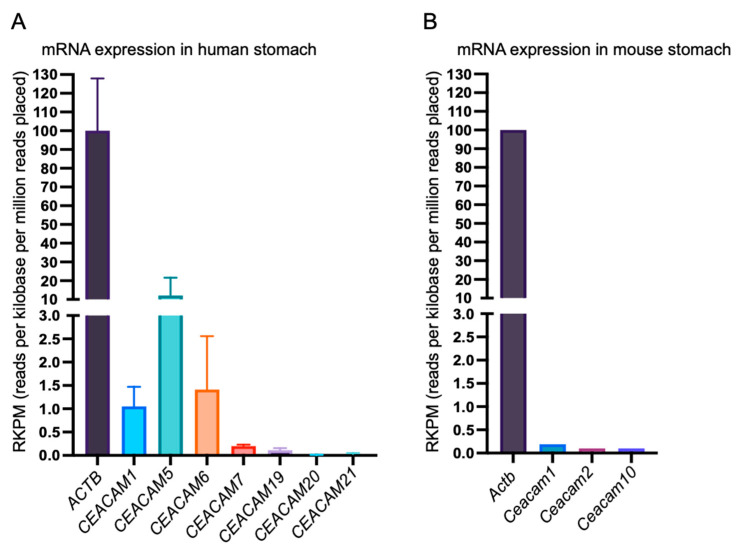
Relative expression levels of human and mouse mRNAs encoding membrane CEACAMs (human) and membrane Ceacams (mouse) in the stomach. Relative levels of human *CEACAM1*, *CEACAM5*, *CEACAM6*, *CEACAM7*, *CEACAM19*, *CEACAM20*, and *CEACAM21* mRNA expression compared to *ACTB* (*β-ACTIN* mRNA) in human stomach (**A**). Relative levels of mouse *Ceacam1*, *Ceacam2*, and *Ceacam10* mRNA compared to *Actb* (*β-Actin*) mRNA in mouse stomach (**B**). It should be noted that the mouse genome does not contain human *CEACAM5*, *CEACAM6*, and *CEACAM7* orthologues, whereas the human genome does not contain a mouse *Ceacam2* orthologue.

Quantitative analysis of mRNA expression for epithelial CEACAMs/Ceacams in the stomach using a public database [NCBI Gene, https://www.ncbi.nlm.nih.gov/gene/] (accessed on 15 December 2021) revealed that the expression of mouse *Ceacam1* was 5-to-10-fold less than that of human *CEACAM1* (Figure 1) [28,29]. The absence of genes encoding GPI-anchored Ceacams in the mouse genome [25,27] indicates that there is no expression of mouse orthologues of human CEACAM5 and CEACAM6 in the mouse stomach. Whereas *Ceacam1* has a duplicated homologue *Ceacam2* in the mouse genome, its mRNA was detected at a very low level in the alimentary tract (Figure 1). Thus, Ceacam1 may be almost all of the epithelial Ceacams expressed in mouse gastric epithelial cells. Together with the results of previous studies showing that *H. pylori* HopQ does not significantly bind to mouse Ceacam1 [22,23] and the fact that mice do not possess GPI-anchored membrane Ceacams such as those corresponding to human CEACAM5 and CEACAM6, the finding raises the possibility that *H. pylori* interacts much less efficiently with mouse gastric epithelial cells than with human epithelial cells because of the low expression level of Ceacam1 with markedly reduced HopQ-binding activity, which dampens *H. pylori* T4SS-mediated CagA delivery.

**Figure 2:**
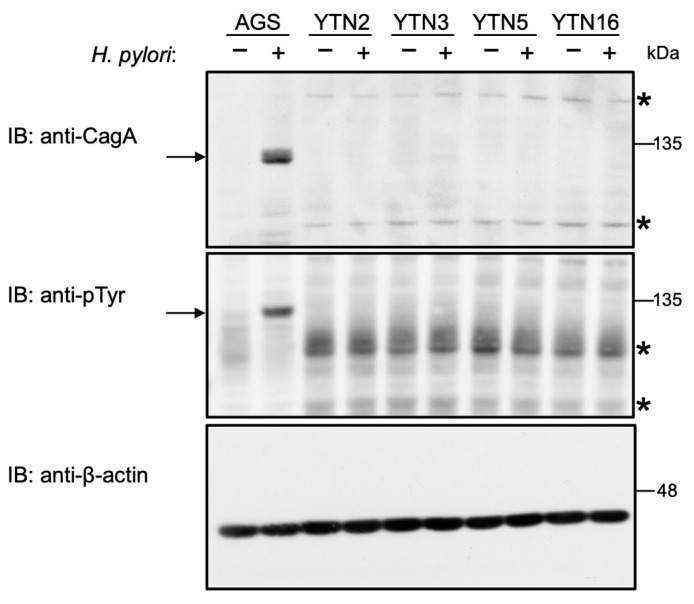
Infection with *cagA*-positive *H. pylori* is not able to deliver CagA into mouse gastric epithelial cells. Mouse gastric epithelial cells (YTN2, YTN3, YTN5, and YTN16) and AGS human gastric epithelial cells were infected with *H. pylori* NCTC11637 strain for 7 h at a MOI of 100. The cells were harvested and analyzed for CagA and tyrosine phosphorylation (pTyr) of CagA proteins. CagA is detectable at 135 kDa as indicated by the arrow in the anti-CagA and anti-pTyr immunoblot. Asterisks (*) denote the non-specific band observed for cell lysates of mouse origins. Representative image of two independent experiments. Experiments were carried out in biological duplicates (*n* = 2) and the representative images are shown.

To test the above-described possibility, we used four mouse gastric epithelial cell lines—YTN2, YTN3, YTN5 and YTN16—that were established from gastric carcinoma induced by treating C57BL/6 mice with *N*-methyl-*N*-nitrosourea (MNU) [30]. Consistent with the mRNA expression profile in the stomach (Figure 1), immunoblotting analysis using an anti-CEACAM1 antibody, which recognizes both human CEACAM1 and mouse Ceacam1, revealed that the expression level of Ceacam1 in mouse gastric epithelial cells was much lower than the expression level of CEACAM1 in AGS human gastric cancer-derived epithelial cells (Supplementary Figure S1). We next conducted an in vitro infection experiment using gastric epithelial cells infected with the *H. pylori cagA*-positive Western standard strain NCTC11637 strain. (Hereafter “*H. pylori”* denotes the NCTC11637 *cagA* positive strain unless otherwise stated.) Since delivered CagA undergoes tyrosine phosphorylation by host cell kinases [31,32], the level of tyrosine-phosphorylated CagA represents the amounts of CagA delivered into gastric epithelial cells by *H. pylori.* As in many other studies, AGS cells were used as positive control cells for the infection experiment. Since a CagA delivery experiment is usually performed by incubation of gastric cells with *H. pylori* for 3–7 h, during which the amount of delivered CagA is proportional to the duration of incubation, we used 7 h incubation to see maximal CagA delivery by the bacterial infection. At 7 h after the onset of *H. pylori* infection, cell lysates were prepared from infected gastric epithelial cells and subjected to immunoblotting with an anti-CagA antibody that detects both CagA derived from host cell surface-attached *H. pylori* as well as CagA delivered into host cells and with an anti-phosphotyrosine (pTyr) antibody that specifically detects host cell-delivered CagA. The results of the experiment showed that a substantial amount of *H. pylori* was attached to the surface of human gastric epithelial cells, to which CagA was efficiently delivered into the cells, as determined by the tyrosine-phosphorylated CagA band. In striking contrast, only a small amount of *H. pylori* was attached to and only a small amount of CagA was delivered into mouse gastric epithelial cells (Figure 2). The same results were reproducibly obtained when another *H. pylori cagA*-positive strain (G27) was used for the infection experiment (Supplementary Figure S2). The results indicated that, in contrast to the case of human gastric epithelial cells, *H. pylori* neither associated stably with mouse gastric epithelial cells nor efficiently delivered CagA into mouse gastric epithelial cells.

### 2.2. The CagA Protein Is Efficiently Translocated and Phosphorylated in Human CEACAM-Expressing Mouse Gastric Epithelial Cells

**Figure 3:**
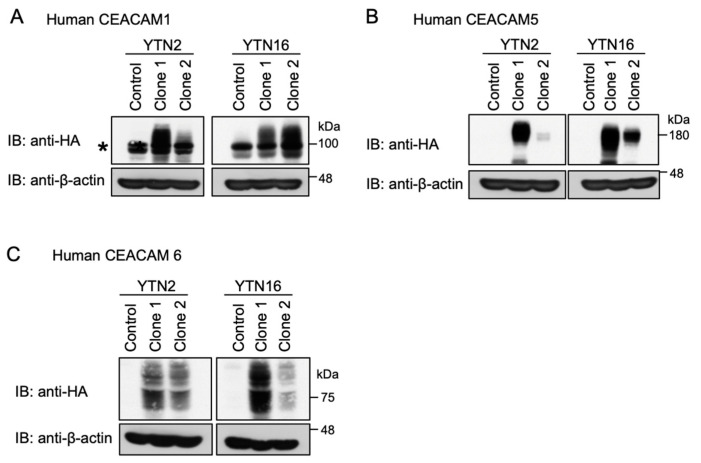
Establishment of human CEACAM-expressing mouse gastric epithelial cells. (**A**) YTN2- and YTN16-derived hemagglutinin (HA)-tagged human CEACAM1 stable cells (clone 1 and clone 2) were harvested and analyzed for expression. Parental cell lines were used as control. The asterisk (*) denotes the non-specific band observed by immunoblotting of YTN2 and YTN16 mouse cell lysates with a human CEACAM1 antibody. (**B**) YTN2- and YTN16-derived HA-tagged human CEACAM5 stable cells (clone 1 and clone 2) were harvested and analyzed for expression. Parental cell lines were used as control. (**C**) YTN2- and YTN16-derived HA-tagged human CEACAM6 stable cells (clone 1 and clone 2) were harvested and analyzed for expression. Parental cell lines were used as control. CEACAMs are recognized as broad bands as they are heavily glycosylated in the cells.

To examine whether impairment of CagA delivery into mouse gastric epithelial cells by *H. pylori* was due to the weak interaction of *H. pylori* HopQ with mouse Ceacams both qualitatively and quantitatively, we established transfectant cells that stably express hemagglutinin (HA)-tagged human CEACAM1 (the CEACAM1-4s isoform), human CEACAM5, or human CEACAM6 using two mouse gastric epithelial cell lines—YTN2 and YTN16—which were arbitrarily chosen from the four mouse gastric epithelial cell lines as they displayed similar morphology and growth rates (Figure 3). The broad bands corresponding to human CEACAM1, 5, and 6 shown in immunoblotting were consistent with the notion that they were heavily glycosylated by post-translational modification.

**Figure 4:**
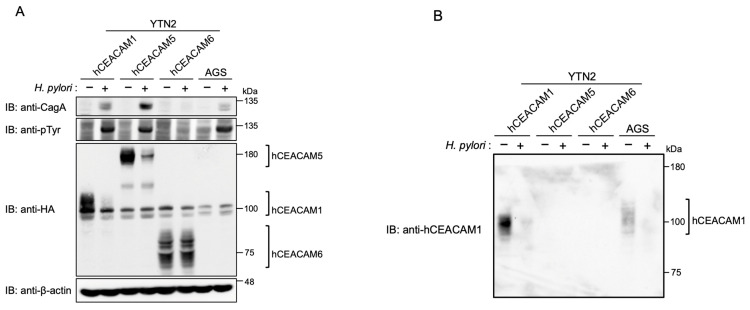
Infection with *cagA*-positive *H. pylori* is capable of delivering CagA into mouse gastric epithelial cells expressing human CEACAM. YTN2 cell-derived hemagglutinin (HA)-tagged human CEACAM1 (hCEACAM1), human CECAM5 (hCEACAM5), and human CEACAM6 (hCEACAM6) stable cells and AGS cells were infected with *H. pylori* NCTC11637 for 7 h at a MOI of 100. The cells were harvested and analyzed for CagA, tyrosine phosphorylation (pTyr) of CagA, and HA-tagged CEACAM proteins. The broad bands represent heavily glycosylated bands of CEACAMs (**A**). The anti-HA membrane was reprobed with an antihuman CEACAM1 (**B**). Experiments were carried out in biological duplicates (*n* = 2) and the representative images are shown. See also Supplementary Figures S3–S5.

The YTN2 or YTN16 cell-derived transfectants stably expressing human CEACAM1 were then infected with *H. pylori*. Ectopic expression of human CEACAM1 in mouse gastric epithelial cells made it possible for *cagA*-positive *H. pylori* to deliver CagA into the cells as determined by tyrosine-phosphorylated CagA (Figure 4A, Supplementary Figure S3). Likewise, stable expression of human CEACAM5, which does not have a mouse orthologue, enabled CagA delivery into mouse gastric epithelial cells by *cagA*-positive *H. pylori* infection (Figure 4A, Supplementary Figure S4). In the infection study, we detected a larger amount of CagA proteins in lysates prepared from YTN2-derived cells stably expressing CEACAM1 (YTN2/hCEACAM1) or CEACAM5 (YTN2/hCEACAM5), possibly due to increased binding of *H. pylori* to the surface of mouse gastric epithelial cells expressing these human CEACAM proteins (Figure 4A, Supplementary Figures S3 and S4), providing additional evidence that both human CEACAM1 and human CEACAM5 efficiently bind to *H. pylori* HopQ. Since *H. pylori* HopQ also interacts with human CEACAM6 in vitro [22,23], we also generated stable human CEACAM6 transfectants from YTN2 or YTN16 cells as parental cells and infected them with *H. pylori* for 7 h. Despite sufficient expression of human CEACAM6 on the stable transfectants, there was no evidence for CagA delivery (Figure 4A, Supplementary Figure S5). From these observations, we concluded that *H. pylori* CagA can be translocated and tyrosine-phosphorylated into mouse gastric epithelial cells stably expressing human CEACAM1 or human CEACAM5 but not into cells stably expressing human CEACAM6. In this regard, it should be noted that the AGS cell lysates used for positive controls (third panel from the top) did not display bands corresponding to human CEACAM1, 5, and 6. This was simply because the immunoblotting experiment was carried out by using an anti-HA antibody that detects ectopically expressed human CEACAMs but not endogenous human CEACAMs. Indeed, stripping and reprobing of the same filter with an antihuman CEACAM1 antibody revealed the presence of endogenous CEACAM1 in AGS cells (Figure 4B).

### 2.3. HopQ Interaction Transiently Reduces the Cellular Levels of Cellular CEACAM Protein

**Figure 5:**
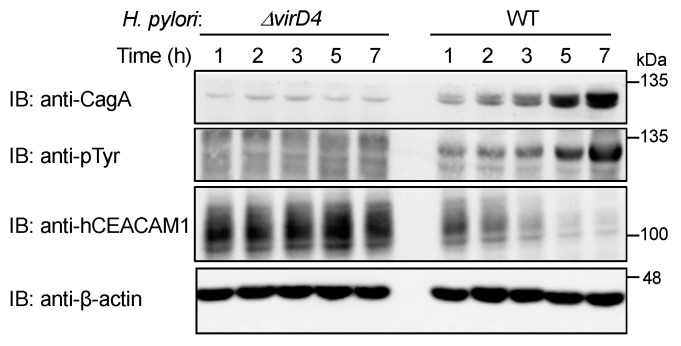
*H. pylori* T4SS contributes to deduction of the level of cellular CEACAM protein. AGS cells were infected with wild-type (WT) *H. pylori* or isogenic *ΔvirD4* strain for consecutively 7 h at a MOI of 100. The cells were harvested and analyzed for CagA, tyrosine phosphorylation (pTyr) of CagA, and human CEACAM1 (hCEACAM1) proteins. Experiments were carried out in biological duplicates (*n* = 2) and the representative images are shown.

During the course of the *H. pylori* infection experiment, we noticed that the level of ectopically expressed human CEACAM1 or CEACAM5 was substantially diminished after 7 h of infection with wild-type *H. pylori* (Figure 4A, indicated by comparison of lane 1 and lane 2 for human CEACAM1 and by comparison of lane 3 and lane 4 for human CEACAM5). Along with ectopic expression, endogenous CEACAM1 expression was also reduced (Figure 4B; lane 7 and lane 8). In contrast, there was no decrease in the level of human CEACAM6 at 7 h after *cagA*-positive *H. pylori* infection (Figure 4A, indicated by comparison of lane 5 and lane 6). Hence, the reduction in human CEACAM was correlated with its ability to bind to *H. pylori* HopQ and subsequent delivery of CagA into host cells. Given this, we next investigated the relationship between the functional T4SS and reduction in human CEACAM1 expression. For this purpose, we infected AGS cells with wild-type *H. pylori* and its isogenic *ΔvirD4* strain that lack the functional T4SS (Figure 5). The results of the experiment revealed that tyrosine phosphorylation of CagA was detectable within 1 h after wild-type *H. pylori* infection. Reduced CEACAM1 expression was also detected within 1 h after infection and the magnitude of CEACAM1 reduction was correlated with elevated levels of CagA tyrosine phosphorylation in a time-dependent manner (Figure 5). In AGS cells infected with the *ΔvirD4* isogenic strain, the level of CEACAM1 expression was stable and constitutive during 7 h infection. Thus, a reduction in human CEACAM1 expression was substantially dependent on the presence of the functional T4SS (Figure 5).

**Figure 6:**
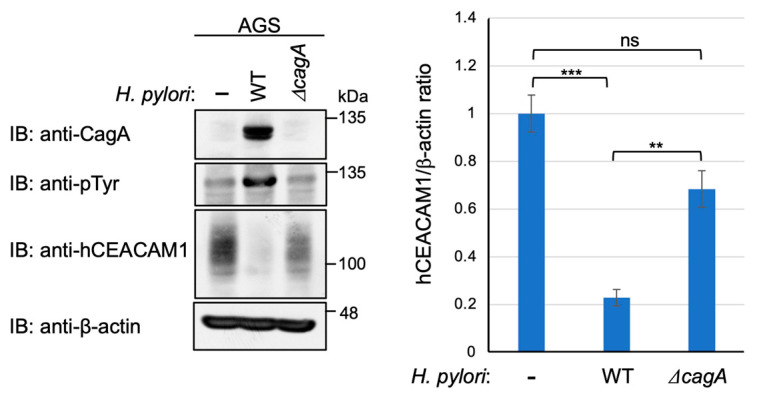
CEACAM-mediated CagA delivery reduces the level of cellular CEACAM protein. AGS cells were infected with wild-type (WT) *H. pylori* or isogenic *ΔcagA* strain for 7 h at a MOI of 100. The cells were harvested and analyzed for the expression of total CEACAM1 protein. Experiments were carried out in biological triplicates (*n* = 3) and the representative images are shown (**left**). Quantification of the intensity of CEACAM1 relative to β-actin (**right**). Error bars indicate mean ± SD, *n* = 3. Data were analyzed by one-way ANOVA and the Bonferroni post hoc test. *** *p* < 0.001, ** *p* < 0.01, ns; non-significant.

**Figure 7:**
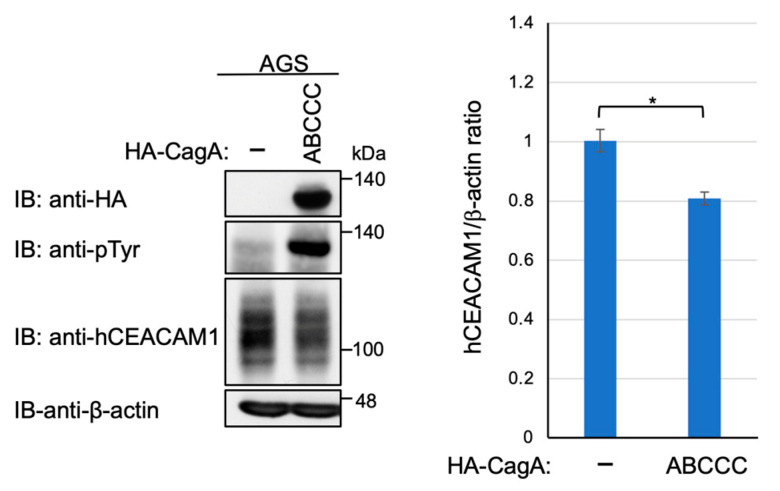
Plasmid-mediated expression of CagA is only a modest reduction in the level of cellular CEACAM protein. AGS cells were transfected with HA-tagged CagA expression vector. The cell lysates were subjected to immunoblotting with indicated antibodies. Experiments were carried out in biological triplicates (*n* = 3) and the representative images are shown (**left**). Quantification of the intensity of CEACAM1 relative to β-actin (**right**). Error bars indicate mean ± SD, *n* = 3. * *p* < 0.05.

We then investigated the possibility that CagA delivered into gastric epithelial cells via the functional T4SS generates a signal that downregulates CEACAM1 or CEACAM5 expression. To test this idea, we infected AGS cells with wild-type *H. pylori* or its isogenic strain lacking the *cagA* gene (*ΔcagA*). The results of the experiment revealed a slight increase in the level of human CEACAM1 expression upon infection with the *ΔcagA* strain (Figure 6). Similar results were observed in Kato-III cells (Supplementary Figure S6). Reciprocally, ectopic expression of cDNA-delivered CagA in AGS cells gave a slight reduction in the level of human CEACAM1 (Figure 7). These results indicated that the functional T4SS plays a major role in the reduction in CEACAM1 and CEACAM5 by *H. pylori* infection, although the contribution of delivered CagA was marginal if any. In addition, to evaluate the time span of reduction in CEACAM1 expression upon *H. pylori* infection, AGS cells and Kato-III cells were infected with *cagA*-positive *H. pylori* for time frames of 0, 7, and 24 h, and CEACAM1 expression was evaluated in the above-mentioned time frames of infection. It was observed that the expression level of CEACAM1 decreased at 7 h as observed previously, which was restored by 24 h of infection (Supplementary Figure S7), suggesting that the expression of CEACAM1 oscillates throughout the infection period.

## 3. Discussion

We found in the present study that, in stark contrast to human gastric epithelial cells, mouse gastric epithelial cells resist T4SS-mediated CagA injection by *H. pylori cagA*-positive strains. Though not natural hosts of *H. pylori*, mice have been routinely used for in vivo studies of *H. pylori* infection. However, in most cases, *H. pylori*-infected mouse only develop lymphocytic gastritis without progression to severe gastric lesions including gastric cancer [33,34,35]. Only a few *cag*PAI-positive strains have been successfully adapted for long-term colonization in the mice stomach and infection of mice with these strains can lead to the development of chronic active gastritis with neutrophil infiltration, progressing to atrophy and metaplasia [36,37]. Nevertheless, even long-term infection of mice with such *cagA*-positive strains does not spontaneously induce gastric cancer, indicating that mice do not faithfully phenocopy gastric lesions induced by chronic *cagA*-positive *H. pylori* infection in human. Since transgenic expression of CagA in mouse gastric epithelial cells gave rise to the induction of gastric cancer [17,38], the failure of gastric cancer development in the stomach of mice infected with *cagA*-positive *H. pylori* does not seem to be due to the insensitivity of mouse gastric epithelial cells to the oncogenic action of CagA. Instead, the results of the present study revealed that *cagA-*positive *H. pylori* failed and is incapable of delivering the CagA oncoprotein into mouse gastric epithelial cells.

**Figure 8:**
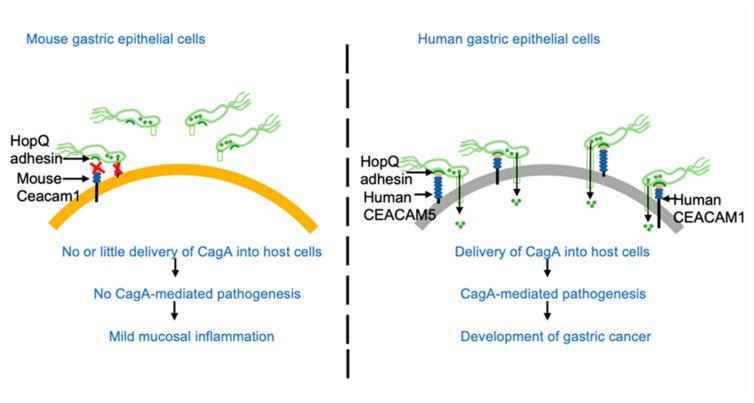
Schematic representation of *H. pylori* infection in mouse and human gastric epithelial cells. Due to low expression of mouse Ceacam1 followed by the incompatible interaction between mouse Ceacam1 and *H. pylori* HopQ, there is no or little delivery of CagA in the mouse gastric epithelial cells. In contrast, compatible binding of *H. pylori* HopQ to expressed human CEACAMs leads to efficient delivery of CagA in human gastric epithelial cells leading to CagA mediated pathogenesis.

It has been shown that the *H. pylori* adhesin HopQ is capable of strongly interacting with several CEACAM proteins including CEACAM1, CEACAM3, CEACAM5, and CEACAM6 and that the HopQ-CEACAM interaction is crucial for T4SS-mediated delivery of CagA by *H. pylori*. Since human CEACAM3 is specifically expressed on granulocytes [26], it does not seem to play a substantial role in the delivery of CagA into gastric epithelial cells. Previous studies also showed that the HopQ-binding ability of mouse Ceacam1 is much less than that of human CEACAM1 (if any) [22,23]. In the present study, we found that the mouse stomach expresses only a small amount of Ceacam1. Although mice possess Ceacam2, a Ceacam1 homologue (80% similarity) that is absent in humans, Ceacam2 is expressed at a very low level in the mouse stomach [29]. Together with the fact that the mouse genome does not possess Ceacam5 and Ceacam6 genes [24,25], the present study revealed that epithelial cells in the mouse stomach do not express Ceacams that are capable of strongly binding with *H. pylori* HopQ both qualitatively and quantitatively. As a consequence, while *cagA*-positive *H. pylori* binds to the surface of mouse gastric epithelial cells via adhesins such as BabA, SabA, and LabA, their weak interactions do not allow substantial CagA delivery into mouse gastric epithelial cells via the T4SS in the absence of HopQ–Ceacam interaction (Figure 8).

A previous study showed that *H. pylori* binds to the membrane surface of Chinese hamster ovary (CHO) cells ectopically expressing human CEACAM1 in a HopQ-dependent manner [23]. However, it was not examined in that study if CagA is delivered into human CEACAM1-expressing CHO cells. Ectopic expression of human CEACAM1, CEACAM5 or CEACAM6 in human embryonic kidney 293 (HEK293) cells reconstituted the HopQ/CEACAM interaction, which enabled translocation of *H. pylori* CagA into HEK293 cells [22,23]. The results of the present study also revealed that, although *cagA*-positive *H. pylori* failed to deliver CagA into mouse gastric epithelial cells, ectopic expression of a single human epithelial CEACAM such as CEACAM1 or CEACAM5 enables *H. pylori*-mediated CagA delivery in mouse gastric epithelial cells, the bona fide targets of *H. pylori*. The results indicated that both membrane-spanning CEACAMs and GPI-anchored membrane CEACAMs are capable of provoking CagA delivery upon binding with HopQ. On the other hand, ectopic expression of another GPI-anchored CEACAM, CEACAM6, did not support *H. pylori*-mediated CagA delivery into mouse gastric epithelial cells. Since HopQ binds to recombinant CEACAM6 in vitro with high affinity [22,23], the results of the present study indicate that GPI-anchored CEACAM6 may cause an allosteric change in the IgV-like N-terminal domain that dampens the HopQ–CEACAM6 interaction. Collectively, these observations indicated that the lack of stable interaction between *H. pylori* HopQ and mouse Ceacams, which dampens CagA delivery into gastric epithelial cells, is responsible for the observed differences in pathological changes in the stomach between mice and humans infected with *cagA*-positive *H. pylori* [33,34,35,39]. 

The level of tyrosine-phosphorylated CagA, a surrogate marker of host cell-delivered CagA, in mouse gastric epithelial cells that stably express human CEACAM1 or CEACAM5 was comparable to that of CagA in AGS cells upon infection with *cagA*-positive *H. pylori.* Consistently, a substantially larger amount of CagA was detected in cell lysates prepared from gastric epithelial cells ectopically expressing human CEACAM1 or CEACAM5 than in cell lysates prepared from parental mouse gastric cells, indicating enhanced *H. pylori* adhesion to the mouse cells via ectopically expressed human CEACAM1 or CEACAM5. 

Aside from the role of HopQ–CEACAM interaction in CagA delivery, we also found a time-dependent reduction in the cellular level of human CEACAM1 following *cagA*-positive *H. pylori* infection (Figure 5). The presence of the functional T4SS but not CagA delivery is critical for CEACAM reduction. A reduction in human CEACAM1 in human gastric epithelial cells infected with *H. pylori* was observed for both endogenous and exogenous CEACAMs, suggesting that the downregulation occurs at the post-transcriptional level. Since the infection-associated CEACAM reduction, shown at 7 h after infection, was restored at 24 h after infection, the HopQ–CEACAM interaction provokes proteolytic cleavage of the extracellular domains of CEACAMs, which causes shedding or degradation of HopQ-bound CEACAMs. Whether the decreased HopQ–CEACAM interaction is beneficial to the host or bacterium is an important issue to be considered. The CEACAM degradation/cleavage might be advantageous for *H. pylori* as it would enable detachment of *H. pylori* from CagA-injected cells and movement to neighborhood epithelial cells as new targets. Alternatively, decreases in membrane-associated CEACAMs in *H pylori*-infected gastric epithelial cells may generate a negative feedback loop for the CagA pathogenic action of CagA, which prevents excess CagA delivery into a single epithelial cell that may provoke premature cell senescence or genomic instability depending on the functional status of p53 [16,40]. Molecular identification of the CEACAM proteases, which can be of human or bacterium origin, may provide a clue to understand why the *H. pylori* HopQ–human CEACAM1/5 interaction undergoes transient reduction during infection of *H. pylori* carrying the functional T4SS. 

The repetitive expansion of CEACAM genes in primates and rodents has been assumed to be associated with positive selection [41,42]. Previous analyses have reported CEACAM genes encompass a large number of nonsynonymous Single Nucleotide Polymorphisms (SNPs) and Copy Number Variations (CNVs) with these variations having high differentiation in the population [43]. The genetic diversity in CEACAM has been reported as a probable factor of human susceptibility to meningococcal disease [44]. The diversity in the CNVs and SNPs may also influence *H. pylori* binding affinity and the T4SS-mediated delivery of CagA. Future investigation on the relationship between variations in CEACAMs in humans and the ability of CagA injection could explain the variability in *H. pylori* infection and development of gastric cancer in the infected populations. 

In summary, our study has revealed the mechanisms underlying species specificity in the pathogenicity of *H. pylori*. To investigate the role of CagA in gastric carcinogenesis using the mouse as an in vivo model of *H. pylori* infection, it should be important to specifically express human epithelial CEACAMs in mouse gastric epithelial cells because ectopic expression of CEACAMs in multiple distinct cell types, including immune cells, might artificially modify gastric lesions induced by chronic *cagA*-positive *H. pylori* infection.

## 4. Materials and Methods

### 4.1. Expression Vectors

The cDNAs encoding C-terminally hemagglutinin (HA)-tagged human CEACAM 1–4s, N-terminally HA-tagged human CEACAM5 and human CEACAM6 were subcloned into a pcDNA3 vector. HA-tagged wild-type *cagA* derived from *H. pylori* NCTC11637 was cloned into the pSP65SRα mammalian expression vector [45].

### 4.2. Cell Culture and Transfection

AGS and Kato-III human gastric cancer cells were purchased from American Type Culture Collection (ATCC). YTN mouse gastric cancer cells were described previously [30]. AGS and Kato-III cells were cultured in RPMI 1640 containing 10% fetal bovine serum (FBS). YTN cells (YTN2, YTN3, YTN5, and YTN16) were cultured in DMEM containing 10% FBS and glucose 4500 mg/L using collagen type I-coated culture dishes. AGS cells and YTN16 cells were transfected with DNA using Lipofectamine 2000 reagent (Invitrogen, Carlsbad, CA, USA). YTN2 cells were transfected with DNA using Lipofectamine and PLUS reagent (Invitrogen, Carlsbad, CA, USA).

### 4.3. Helicobacter Strains and Culture

*Helicobacter pylori* standard strain NCTC11637 and its isogenic strains lacking the *cagA* gene (*ΔcagA*) and lacking the *virD4* gene (*ΔvirD4*) have been described previously [45,46]. *H. pylori* strains (NCTC11637 and G27) were grown in Brucella Broth supplemented with 10% FBS and incubated in a microaerobic atmosphere by using AnaeroPack helico (Mitsubishi Gas Chemical, Tokyo, Japan) at 37 °C.

### 4.4. Establishment of Stable Cell Lines

YTN2 and YTN16 cells were transfected with linearized hemagglutinin (HA)-tagged CEACAM expression vector (pcDNA3-CEACAM) together with linearized pBABE-puro vector. At 24 h post transfection, cells were incubated in culture medium containing puromycin, 8 μg/mL for YTN2 cells, and 12 μg/mL for YTN16 cells, respectively. Puromycin-resistant colonies were picked up with cloning rings and then each colony was evaluated for the expression of CEACAM by immunoblotting.

### 4.5. Immunoblot Analysis

Cells were harvested and lysed in RIPA buffer (150 mM NaCl, 0.1% SDS, 0.5% sodium deoxycholate, 50 mM Tris-HCl pH 7.5, 1 mM EDTA, 2 mM Na_3_VO_4_, 10 mM NaF, 10 mM β-glycerophosphate, 2 mM PMSF, 10 μg/mL leupeptin, 10 μg/mL trypsin inhibitor, 10 μg/mL aprotinin, 1% NP-40). Samples were run on SDS-PAGE gel, and separated proteins were transferred to PVDF membranes (Merck Millipore, Country Cork, Ireland). After transfer, membranes were treated with a blocking solution (1% BSA or 5% non-fat milk in 0.1% Tween 20-TBS). The membranes were incubated with primary antibodies. Anti-β-actin antibody (8H10D10), anti-hemagglutinin (HA) antibody (6E2), anti-CEACAM1 anrtibody (D3R80), and anti-CECAM1 (D1P4T) antibody were obtained from Cell Signaling Technology (Danvers, MA, USA); anti-phosphotyrosine antibody (4G10) was obtained from Millipore (Darmstadt, Germany); anti-HA antibody (3F10) was obtained from Roche (Mannheim, Germany), and anti-CagA antibody (HPP-5003-9) was obtained from Austral Biologicals (San Ramon, CA, USA). Secondary antibodies used were conjugated with horseradish peroxidase (HRP). Protein bands were visualized by Western blot chemiluminescence reagent (Perkin Elmer Life Sciences, Waltham, MA, USA).

### 4.6. Statistical Analysis

Microsoft Excel (Redmond, WA, USA) and Graph Pad Prism software (San Diego, CA, USA) were used for statistical analysis. Data were analyzed with one-way ANOVA and Bonferroni post hoc test. P values were determined by Student’s t-test. All of the statistical details were stated in the figure legends. Experiments were performed in biological triplicates and representative images are displayed unless otherwise stated.

## Data Availability

The data presented in this study are all contained within the main manuscript and the Supplementary Figures of this article.

## Supplementary Materials

The following are available online at https://www.mdpi.com/article/10.3390/ijms23052492/s1.
