# Supplementary material for: Mouse Gastric Epithelial Cells Resist CagA Delivery by the Helicobacter pylori Type IV Secretion System
Source: Int J Mol Sci. 2022 Feb 24;23(5):2492. doi: 10.3390/ijms23052492 (PMC8910101; doi:10.3390/ijms23052492)
Supplement: Supplementary file 1 [file ijms-23-02492-s001.zip › ijms-1539981-supplementary.pdf]

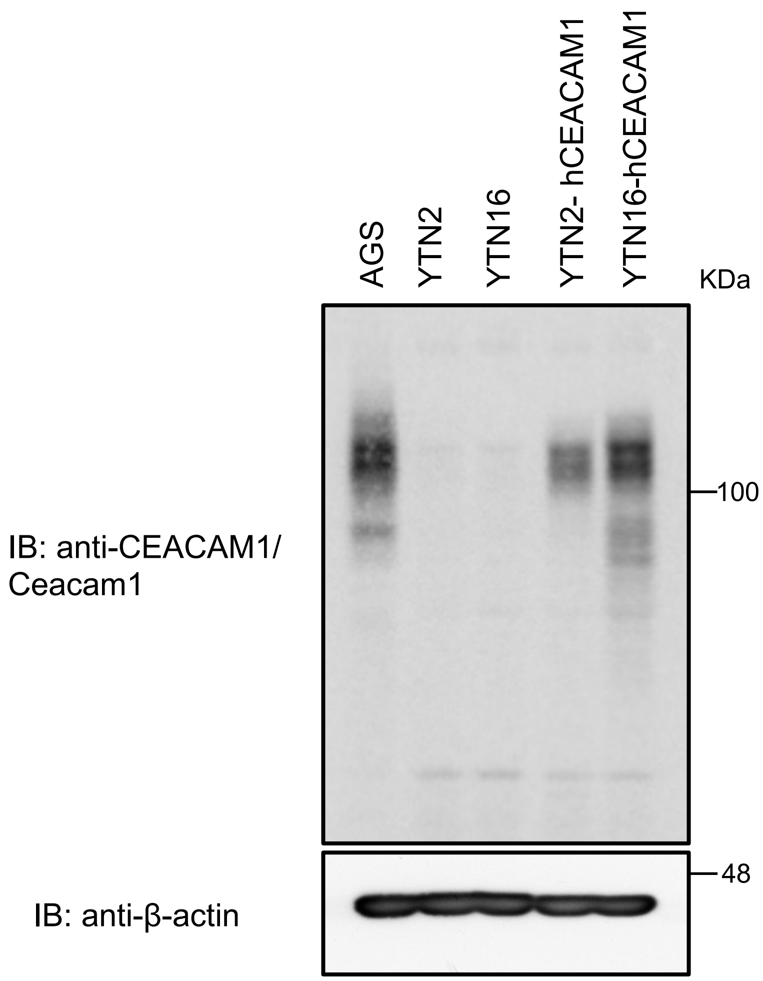

Figure S1. Expression of endogenous CEACAM1 in human and mouse gastric cells. Lysates obtained from human gastric epithelial cell line AGS and mouse gastric cell lines YTN2 and YTN16 were evaluated with monoclonal CEACAM1 antibody specific to both human CEACAM1 and mouse Ceacam1 and was used for comparing the expression between human and mouse. Mouse cells expressing human CEACAM1 (lane 4 and lane 5) was used for comparison of expression.

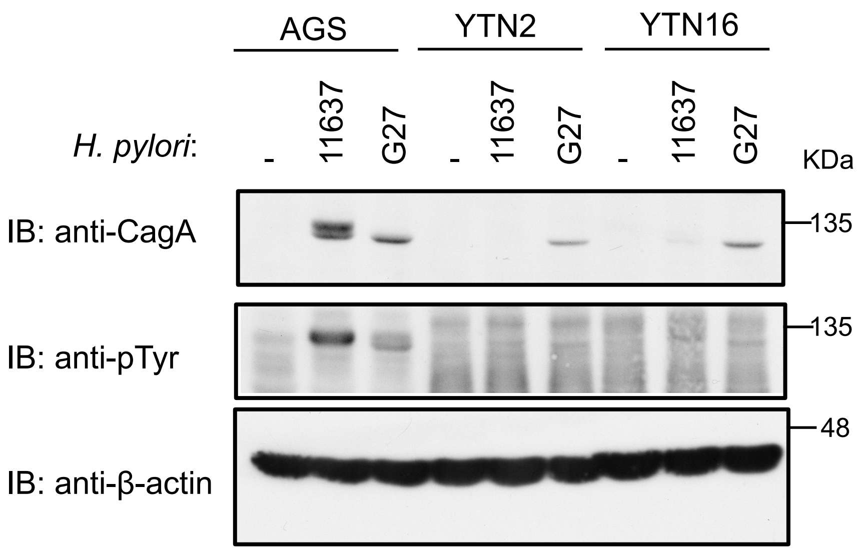

Figure S2 . Infection with *cagA*-positive *H. pylori* is not able to deliver CagA into mouse gastric epithelial cells. Mouse gastric epithelial cells (YTN2, and YTN16) and AGS human gastric epithelial cells were infected with *H. pylori* NCTC11637 and G27 strain for 7 h at a MOI of 100. The cells were harvested and analyzed for CagA and tyrosine phosphorylation (pTyr) of CagA proteins.

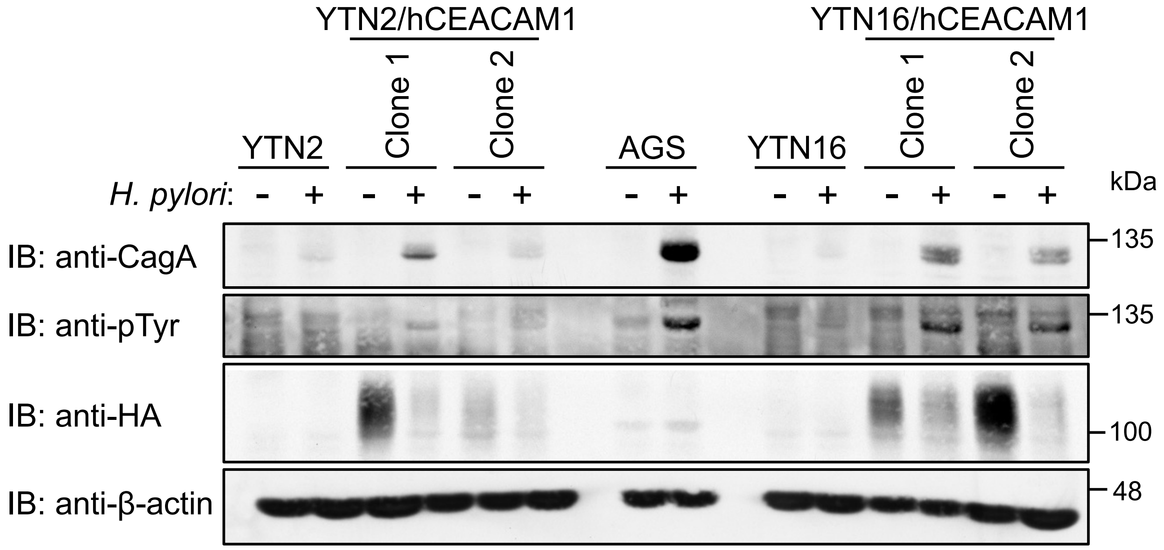

Figure S3. Infection with *cagA*-positive *H. pylori* is capable of delivering CagA into mouse gastric epithelial cells expressing human CEACAM1. YTN2- and YTN16-derived hemagglutinin (HA)-tagged human CEACAM1 stable cells and AGS cells were infected with *H. pylori* NCTC11637 strain 7 h at a MOI of 100. The cells were harvested and analysed for CagA, tyrosine phosphorylation (pTyr) of CagA, and HA-tagged CEACAM1 protein.

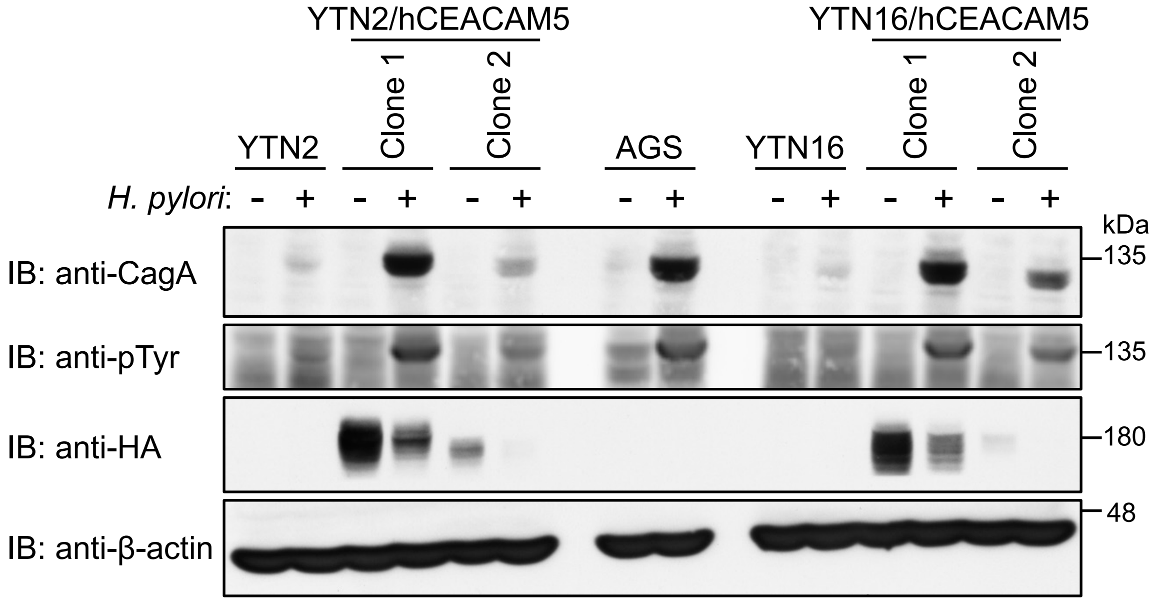

Figure S4. Infection with *cagA*-positive *H. pylori* is capable of delivering CagA into mouse gastric epithelial cells expressing human CEACAM5. YTN2- and YTN16-derived hemagglutinin (HA)-tagged human CEACAM5 stable cells and AGS cells were infected with *H. pylori* NCTC11637 strain for 7 h at a MOI of 100. The cells were harvested and analysed for CagA, tyrosine phosphorylation (pTyr) of CagA, and HA-tagged CEACAM5 protein.

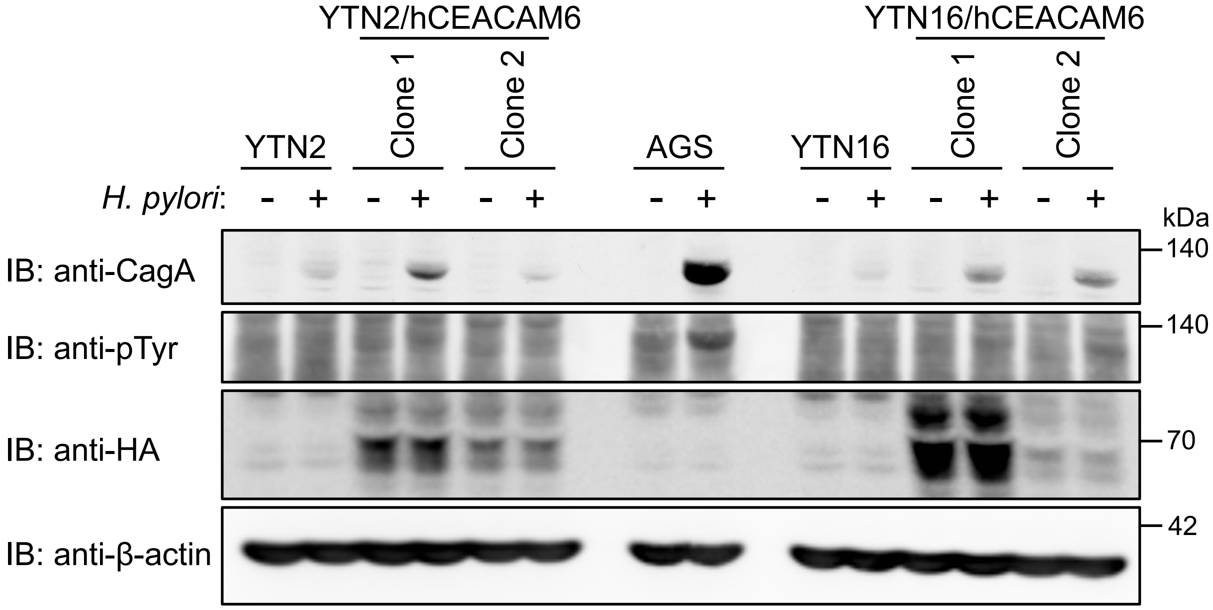

Figure S5. Infection with *cagA*-positive *H. pylori* is not able to deliver CagA into mouse gastric epithelial cells expressing human CEACAM6. YTN2- and YTN16-derived hemagglutinin (HA)-tagged human CEACAM6 stable cells and AGS cells were infected with *H. pylori* NCTC11637 strain 7 h at a MOI of 100. The cells were harvested and analysed for CagA, tyrosine phosphorylation (pTyr) of CagA, and HA-tagged CEACAM6 protein.

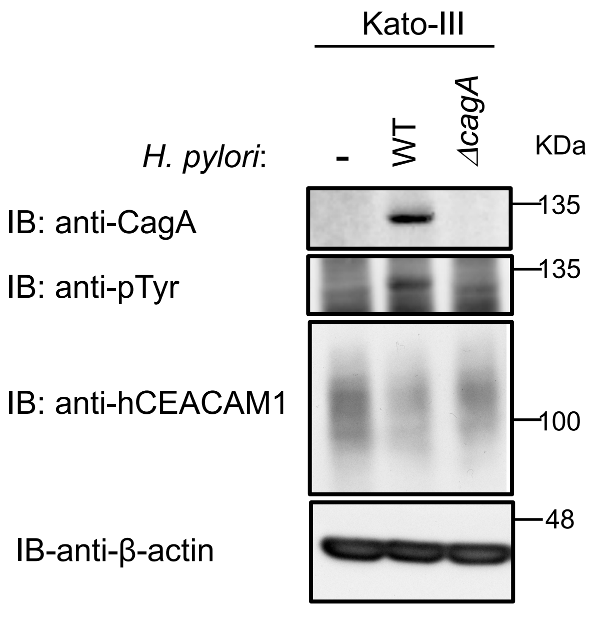

Figure S6: CEACAM-mediated CagA delivery reduces the level of cellular CEACAM protein. Kato-III cells were infected with wild-type (WT) *H. pylori* or isogenic *ΔcagA* strain for 7 h at a MOI of 100. The cells were harvested and analyzed for the expression of total CEACAM1 protein.

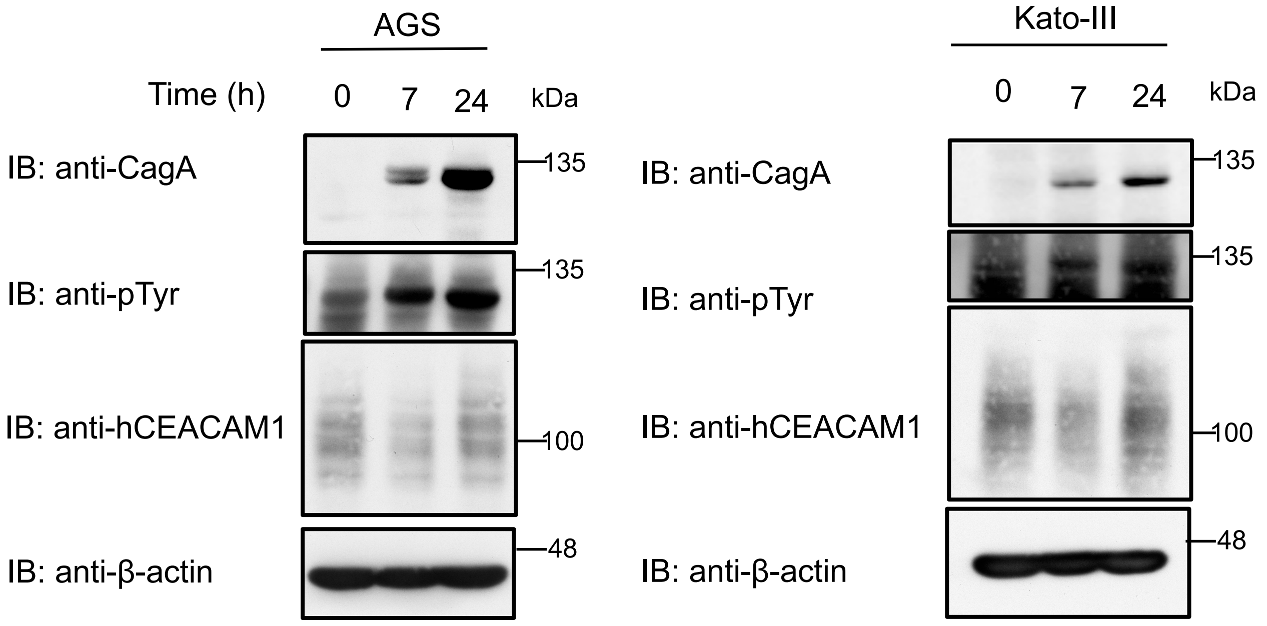

Figure S7. *H. pylori* infection contributes to transient deduction of the level of cellular CEACAM protein. AGS and Kato-III cells were infected with wild-type (WT) *H. pylori* for consecutively 0, 7 and 24 h at a MOI of 100. The cells were harvested and analyzed for CagA, tyrosine phosphorylation (pTyr) of CagA, and human CEACAM1 (hCEACAM1) proteins.
